# Orangutan males make increased use of social learning opportunities, when resource availability is high

**DOI:** 10.1016/j.isci.2024.108940

**Published:** 2024-01-18

**Authors:** Julia Mörchen, Frances Luhn, Olivia Wassmer, Julia A. Kunz, Lars Kulik, Maria A. van Noordwijk, Puji Rianti, Tri Rahmaeti, Sri Suci Utami Atmoko, Anja Widdig, Caroline Schuppli

**Affiliations:** 1 Development and Evolution of Cognition Research Group, Max Planck Institute of Animal Behavior, 78467 Konstanz, Germany; 2 Behavioral Ecology Research Group, Institute of Biology, University of Leipzig, 04103 Leipzig, Germany; 3 Department of Primate Behaviour and Evolution, Max Planck Institute for Evolutionary Anthropology, 04103 Leipzig, Germany; 4 Department of Evolutionary Anthropology, University of Zurich, 8057 Zurich, Switzerland; 5 Institute of Evolutionary Biology of Montpellier (ISEM), University of Montpellier, CNRS, IRD, 34095 Montpellier, France; 6 Comparative Socioecology, Max Planck Institute of Animal Behavior, 78467 Konstanz, Germany; 7 Primate Research Center, Institute of Research and Community Service, IPB University, Bogor 16680, Indonesia; 8 Animal Biosystematics and Ecology Division, Department of Biology, IPB University, Bogor 16680, Indonesia; 9 Department of Biology, Graduate Program, Faculty of Biology and Agriculture, Universitas Nasional, Jakarta 12520, Indonesia; 10 German Centre for Integrative Biodiversity Research (iDiv), Halle-Jena-Leipzig, 04103 Leipzig, Germany

**Keywords:** Nature conservation, Ecology, Zoology

## Abstract

Humans’ colonization of diverse habitats relied on our ancestors' abilities to innovate and share innovations with others. While ecological impacts on innovations are well studied, their effect on social learning remains poorly understood. We examined how food availability affects social learning in migrant orangutan unflanged males, who may learn from local orangutans through peering (i.e., observational social learning). We analyzed 1,384 dyadic associations, including 360 peering events, among 46 wild Sumatran orangutan and 25 Bornean orangutan males, collected over 18 years. Migrants’ peering rates significantly increased with higher food availability and time spent in proximity to others. Furthermore, migrants in the more sociable Sumatran population exhibited significantly higher peering rates compared to the Borneans, suggesting intrinsic and/or developmental effects of food availability on social learning. These findings emphasize the importance of investigating ecological effects on social learning on the immediate, developmental, and intrinsic levels for our understanding of cultural evolution.

## Highlights

• Habitat food availability might affect migrant male orangutans' peering behavior
• On the immediate level, peering rose with food and closeness to local orangutans
• Further migrant males peering rates were higher in the more sociable population
• Thus, food availability shapes social learning on developmental or intrinsic level

## Graphical abstract

**Figure:**
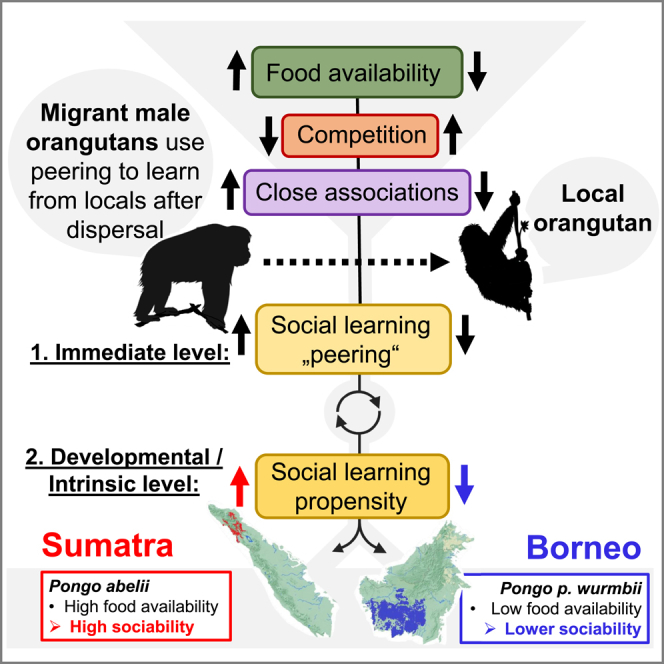

## Introduction

Innovations, i.e., novel, learned behavior patterns acquired by an individual,^1^^,^^2^ are the source of cultural knowledge. Such cultural knowledge is spread and maintained over generations by means of social learning.^3^^,^^4^^,^^5^ Social learning includes any learning that is influenced by the observation of or interaction with another individual.^6^^,^^7^ Culture, hence, at its most basic definition can be described as “socially transmitted innovations,”^1^ and more specifically as the sum of “all behaviors and knowledge that are acquired and passed on within and between generations through social learning.”^8^ Gradual human colonization of an exceptionally wide range of habitats on the planet critically depended on our ancestors' abilities to continuously come up with new behavioral innovations, cumulatively refine them, accumulate modifications over time, and preserve them by sharing them with others via social transmission.^9^^,^^10^^,^^11^^,^^12^^,^^13^^,^^14^ Innovations played a crucial role during early humans’ migration into unknown habitats,^15^^,^^16^ yet they also came at costs such as time and energy investments,^17^ as well as risks in the form of predation and poisoning.^18^^,^^19^ Horizontal social learning from individuals outside of the family/matrilineal/cultural unit, i.e., “strangers,” can lead to the acquisition of new skills and knowledge and thus might have allowed individuals to adapt to new ecological niches, without carrying the costs of individual exploration.^20^^,^^21^ Learning from strangers was thus likely a pivotal element in our species' migratory history.

While the effects of prevailing ecological conditions on innovation rates are well studied in non-human primates, much less is known about such effects on social learning and transmission. Broadly speaking, there are two scenarios on how ecological conditions can affect innovation rates in wild primates: on the one hand, ecological resource availability can modulate individuals’ innovation probabilities via the availability of substrates and materials needed for certain innovations (e.g., hammer and anvil stones needed for tool use, “opportunity hypothesis”^22^) and, on the other hand, ecological resource scarcity may force individuals to come up with new innovations that increase the exploitation of available resources (“necessity hypothesis”^23^^,^^24^). Innovation propensity has been further linked to dispersing individuals, who have a higher need to invent solutions to deal with new problems when leaving familiar natal areas,^14^^,^^25^^,^^26^ but see Schnoell et al.^27^

However, in contrast to individual learning, most forms of social learning require associations between individuals.^7^^,^^28^ Forms of social learning that allow for high-fidelity transmission of information, such as observational social learning (e.g., contextual imitation and production emulation) and interactive social learning,^4^^,^^29^ require close spatial proximity within these associations.^30^ Furthermore, high-fidelity forms of social learning, i.e., being allowed to watch or to interact, may require even higher levels of social tolerance than just being close by.^31^ Because associations and in particular close associations bear significant costs and risks to individuals, ecological conditions may heavily affect social learning opportunities (see in the following). The effect of ecological conditions on horizontal social learning among unrelated adult individuals is expected to be particularly affected by fluctuations in resource availability because, unlike in the parent-offspring context,^32^ social tolerance is not automatically given between individuals that do not share a genetically anchored interest to be socially tolerant of one another,^33^^,^^34^ but see Samuni et al.^35^

Social learning between unfamiliar individuals can entail costs for both the learner and the role model, such as competition over resources,^36^ disadvantages due to competitive information gathering,^37^^,^^38^ accidentally learning wrong or outdated information,^39^ and the increased likelihood of harmful disease transmission, especially the ones from pathogens against which newcomers have not yet developed immunity.^40^^,^^41^^,^^42^^,^^43^^,^^44^ In contrast, mutual benefits of social learning between strangers are the increased likelihood of the exchange of new, valuable knowledge and skills that have critical fitness consequences,^45^^,^^46^ the potential for cooperative relationships,^47^ and the prospect of future mating opportunities.^48^ Accordingly, there is likely strong selective pressure on traits that allow individuals to balance the costs and benefits of associating and social learning from an unfamiliar individual.

Flexible shifting from social to individual learning and back, modulated through ecological resource availability and associated competition over resources, has been studied in the theoretical context through agent-based models^49^ but also in data-based studies, e.g., in wild birds.^50^^,^^51^ These studies found that harsher environments overall favor less reliance on social learning in individuals, likely to avoid resource competition with conspecifics. However, it has also been argued that fluctuation in resource availability during human evolution might have favored the development of tolerant behaviors between nongroup individuals.^34^ Increased propensity for social learning in times of need for knowledge acquisition, such as when dealing with the challenges during migration, may thus significantly improve individuals’ performances and ultimately their survival.^52^^,^^53^^,^^54^ Independent of the ultimate causes of migration or movement in general, individuals that migrate or move a lot have a higher likelihood to experience changes in habitat and social learning opportunities from a wider range of potential role models.^55^^,^^56^^,^^57^ Since geographical distance positively correlates with ecological and cultural differences,^58^ long-distance and long-term movement likely provide the most diverse experiences to individuals. Accordingly, migrating individuals are expected to show increased social learning propensity for optimal resource exploitation and adaptation.

Orangutans provide an ideal study system to investigate how ecological conditions affect social learning: They face substantial energetic constraints because they are large-brained, arboreal, and live in forests with low and fluctuating resource availability.^59^^,^^60^^,^^61^^,^^62^^,^^63^ These constraints are thought to make orangutans’ social tolerance particularly susceptible to the effects of local resource availability.^64^^,^^65^^,^^66^ Accordingly, orangutan sociality is directly linked to the productivity of the habitat they live in: whereas Bornean orangutans live in habitats characterized by low and fluctuating food availability and are semi-solitary, many populations of Sumatran orangutans (particularly the ones in northwest Sumatra) live in forests with higher productivity.^61^ Accordingly, Sumatran populations show higher levels of sociability, in that they spend more time with conspecifics.^67^ Both Sumatran and Bornean orangutans show individual-based fission-fusion social dynamics,^68^ which allows researchers to track opportunities for social learning. In contrast to highly sociable species living in fission-fusion societies like chimpanzees or bonobos,^34^^,^^69^ social learning might be easier to study in semi-solitary species since associations are more overseen and it is thus easier to track who is paying attention to whom (but see limitations part, on potential confounding effects of sociability on social learning patterns). The higher mean association frequency and spatially closer associations of orangutans in northwest Sumatra likely provide individuals with more social learning opportunities from a wider range of conspecifics compared to Bornean individuals. However, how they utilize these social learning opportunities in the wild remains to be investigated. Orangutans are also highly neophobic^19^ and rely heavily on social learning for their skill and knowledge acquisition.^31^^,^^70^^,^^71^ This high reliance on social learning is thought to be the source of their vast and complex cultural repertoires.^58^^,^^72^^,^^73^

As means of social learning, orangutans use i) peering behavior, i.e., the attentive close-range watching of the activities of conspecifics^31^^,^^57^^,^^74^^,^^75^^,^^76^ and ii) food solicitations.^70^^,^^77^ These two means of social learning have been validated in a large range of species, mainly via the contexts in which they occur: they are disproportionally frequently used in learning-intense contexts, directed at knowledgeable individuals, and followed by selective practice of the observed behavior.^31^^,^^57^^,^^78^^,^^79^^,^^80^^,^^81^^,^^82^ Both peering and food solicitations require close spatial proximity between association partners (i.e., social learning opportunities). Not all peering and food soliciting may lead to social learning, as it likely depends on how the peered-at or solicited information relates to the knowledge repertoire of the peering individual. However, peering and food solicitations open a window for knowledge transfer and thus represent likely utilizations of social learning opportunities.

The results of our recent study suggest that adult orangutan migrants use observational social learning in the form of peering to learn about local foods, including complex feeding skills, from knowledgeable resident orangutans.^57^ Generally, adult male orangutans disperse over large distances^83^^,^^84^^,^^85^^,^^86^ which means that during the dispersal process, males likely encounter different types of habitats and host populations.^87^ Mörchen et al. 2023^57^ found that migrant males’ peering decreased with increasing residency (i.e., the duration spent in the area) and thus likely familiarity with the area. Furthermore, migrant males interacted significantly more with the peered-at item after the peering event, than before, providing further evidence that their peering is means to social learning.^57^

### Research aim

Previous studies have shown that increased food availability can lead to increased association time between individuals.^51^^,^^68^^,^^83^^,^^88^ The aim of our study was to investigate how food availability affects horizontal social learning in migrant orangutan males, when associating with resident orangutans. We used a large longitudinal dataset on dyadic associations with and without peering events by 71 migrant unflanged males. These data were collected in the highly sociable population of Sumatran orangutan (*Pongo abelii*) at Suaq Balimbing and in the less sociable population of Bornean orangutan (*Pongo pygmaeus wurmbii*) at Tuanan. Both populations live in peat-swamp forests that differ in terms of food availability due to differences in forest productivity between Sumatran and Bornean forests (see earlier text). These differences in food availability are thought to have led over evolutionary time to differences in social tolerance between the two orangutan populations. Furthermore, on a smaller scale, in both populations, local food availability fluctuates within and throughout the year.^62^^,^^63^

### Rationale and predictions

Ecology may affect social learning on different levels: firstly, prevailing ecological conditions can affect social learning opportunities and the utilization thereof on the immediate proximate level. Thus, we predict that migrant male peering rates will increase with increasing food availability and with increasing time spent in close spatial proximity to peering targets (prediction 1). If the effect of food availability on peering is visible even after correcting for differences in close association (time the peerer and peering target spent within 2 m), we can conclude that food availability affects social learning beyond spatial proximity, likely via effects on social tolerance.

Secondly, ecological conditions experienced during ontogeny may affect the development of social learning propensity and thus result in differences in the tendency to use social learning during later life. Because of the difference in forest productivity between the two populations, males in West Sumatra that dispersed to Suaq experienced significantly higher food availability during development prior to dispersal compared to males at Tuanan, in Borneo. Similarly, the general differences in ecological conditions may affect the genetic fixation of social learning propensity over evolutionary time. Thus, between the populations, we predict that (as a result of developmental or genetic effects, or a combination thereof) migrant males in Suaq will show significantly higher peering rates than migrant males in Tuanan, even when controlling for the prevailing differences in food availability in the study sites, suggesting they make greater use of their social learning opportunities (prediction 2).

## Results

**Figure 1:**
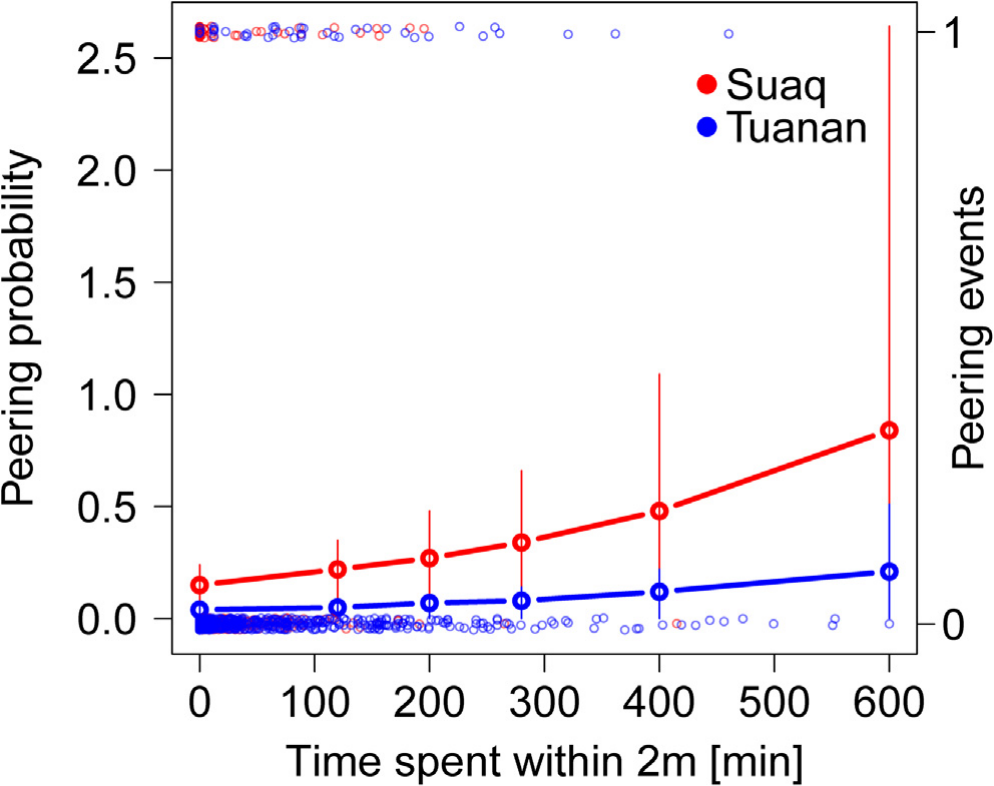
Effects of time spent in close spatial proximity on migrants’ peering Migrant orangutan males’ predicted peering probabilities over the time they spent within 2 m of peering targets. Shown are the model predictions (horizontal lines) and confidence intervals (vertical lines) with all other variables of the model kept constant at their mean. The occurrence of the actual migrant males’ peering events is depicted by transparent smaller open circles with either 0 (no peering) or 1 (peering event) happening, for each daily dyadic association.

**Figure 2:**
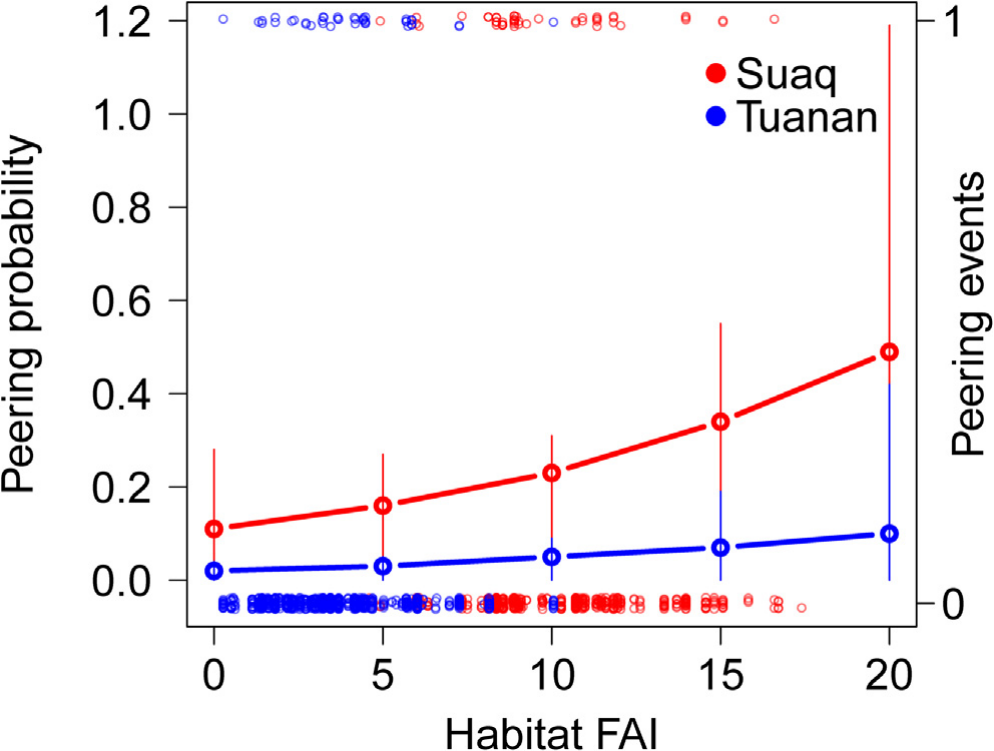
Effects of prevailing food availability on migrants’ peering Migrant orangutan males’ predicted peering probabilities over prevailing habitat food availability, ranging from low (0) to high (20). Shown are the model predictions (horizontal lines) and confidence intervals (vertical lines) with all other variables of the model kept constant at their mean. The occurrence of the actual migrant males’ peering events is depicted by the transparent smaller open circles with either 0 (no peering) or 1 (peering event) happening, for each daily dyadic association. For Suaq the actual FAI values ranged from 3.89 (minimum) to 17.40 (maximum), with a mean FAI value of 10.01. For Tuanan the actual FAI values ranged from 0.27 (minimum) to 10.05 (maximum), with a mean FAI value of 3.76.

**Figure 3:**
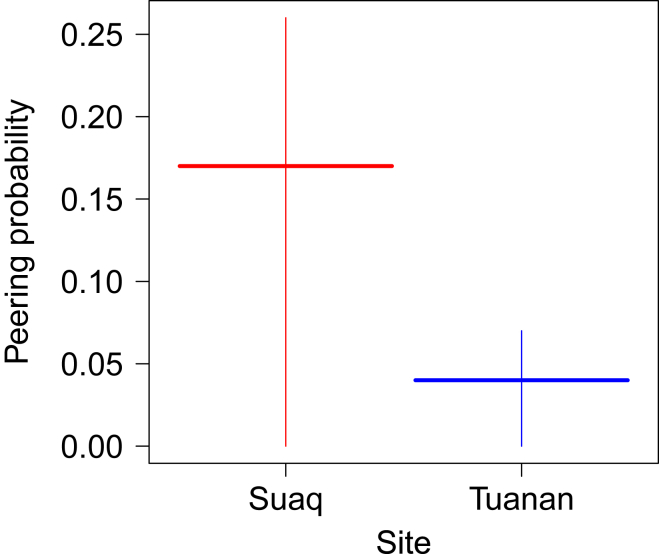
Differences in peering probabilities between the two orangutan populations Migrant orangutan males’ peering probabilities at the more sociable Suaq population in Sumatra (*Pongo abelii*), and at the less sociable Tuanan population in Borneo (*Pongo pygmaeus wurmbii*), with all other variables of the model kept constant at their mean. Shown are the model predictions (horizontal lines) and confidence intervals (vertical lines) for each site.

The comparison between the full and null models showed that as a whole the predictor variables of our model had a significant impact on migrants’ peering rates (likelihood ratio test [LRT]: *X*^*2*^ = 70.79, degrees of freedom [df] = 3, p *<* 0.001). In line with our predictions, we found that first, within populations, migrant orangutan males’ peering rates significantly increased with higher prevailing food availability and the amount of time spent at close spatial distance to residents (Table 1; Figures 1 and 2). Second, we found that migrant orangutan males of the more sociable population of Suaq peer significantly more than the migrant orangutan males in Tuanan (Table 1; Figure 3). In the model we controlled for variables that previously have been shown to affect migrant orangutan males’ peering rates, like the age-sex class of the peering target and the peerer and the number of months a male has been in the study area upon peering date^57^ (Table 2). Furthermore, we controlled for dyad id and year, by including them as random effects (Table 2).

**Table 1:** Effects on migrant orangutan males peering rates

Factor	Factor type	Estimate	SE	Lower CI	Upper CI	χ^2^	df	P
Intercept	Intercept	−0.83	0.22	−7.33	−6.49	–	–	**< 0.001**
Site (Tuanan)	Predictor	−1.4	0.37	−2.16	−0.78	14.63	1	**< 0.001**
FAI	Predictor	0.37	0.16	0.1	0.62	4.45	1	**0.0181**
Time within 2 m	Predictor	0.005	0.002	0.004	0.007	9.08	1	**0.0016**

The results of the GLMM with Poisson family distribution, analyzing the effects of site, FAI, and time spent in close distance of 2 m to the peering target, on migrant males’ peering behavior. Model estimates, standard errors (SEs), confidence intervals (CIs), Chi-square (χ2), and degrees of freedom (df) are provided, based on N = 1,384 daily dyadic observations of migrants associating in distances of 0–50 m with targets of all age-sex classes. The conditional pseudo delta R2 was 0.85. Significant p values are marked in bold font.

**Table 2:** Results of the random effects of Model 1

Groups	Name	Variance	Std.Dev.	N
Age-sex-class party	Present Month Area	0.65	0.8	3
Dyad id	Present Month Area	5.42	2.33	578
Year	Present Month Area	0.28	0.52	18

Shown are variance, standard deviation, sample size of control variables and random slopes of age-sex class of the peering target, dyad id, and year over the number of months the migrant peerer had been present in the study area (“Present Month Area”) at the day of associating with the peering target.

## Discussion

In our study, we looked at the effect of food availability on peering rates as a proxy of observational social learning in two orangutan populations and species that differ in levels of sociability^68^^,^^86^ due to differences in forest productivity.^61^ In contrast to other studies, by investigating peering behavior as an indicator of social learning, we analyzed social learning by directly looking at the moment of potential knowledge transmission, rather than indirectly by studying its potential results.^73^ This allowed us to narrow down the mechanism underlying social transmission. The setup of our study allowed us first to differentiate between the immediate proximate effects of food availability on the utilization of social learning opportunities and second to assess the effects of experienced food availability on the development of social learning propensities, even though differentiating between developmental and genetic effects was not possible.

We found several lines of evidence that ecological conditions modulate observational social learning by dispersing orangutan unflanged males from resident orangutans: First, in line with our predictions, we found that migrant orangutan males’ peering rates significantly increased with increasing local food availability. Further, the migrant orangutan males’ peering rates also significantly increased with increasing time they spent in close spatial proximity to resident orangutans. These results complement the results of previous studies that found that increased food availability can lead to increased association time between individuals.^51^^,^^68^^,^^83^^,^^88^ Strikingly, our results also showed that the positive effect of food availability on the males’ peering persisted even after correcting for the time the males spent in close association, suggesting that food availability might affect the migrants’ social learning rates beyond spatial tolerance. This effect was observed in both populations.

Second, we found that even when controlling for the known study sites’ differences in food availability, migrant orangutan males of the more sociable population of Suaq show significantly higher peering rates than their counterparts in Tuanan, suggesting differences in social learning propensities between migrant males of the two species. All in all, our results suggest that, in addition to increased social learning opportunities provided by more frequent associations,^51^^,^^68^^,^^83^^,^^88^ migrant orangutan males also make increased use of these opportunities when prevailing ecological conditions are favorable.

Similar results have been found in theoretical and empirical studies,^49^^,^^50^ implicating that favorable environments foster social learning, while in harsher environments individuals will shift to individual learning, likely to avoid resource competition with conspecifics. Especially for individuals which do not share a genetically anchored interest to be tolerant to each other, prevailing ecological conditions are likely to affect the levels of social tolerance. This in turn will influence social learning opportunities, the utilization thereof, and ultimately social transmission of information. This means that when learning from unrelated individuals becomes critical, for example after migration,^5^^,^^56^ ecological conditions likely heavily affect individuals’ social learning via modulating the levels of social tolerance and spatial distance between the individuals involved. Mörchen et al. 2023^57^ found that peering rates of migrant males were highest upon arrival in the new area and significantly decreased with increasing time spent in the area. This is consistent with the idea that the “necessity” for socially learning new knowledge is particularly elevated when migrants arrive in a new area. Here we found that, when statistically controlling for the time present in the area, migrants’ peering rates increased in times with increasing food availability. The findings of Mörchen et al. 2023^57^ and the current study showed that the necessity to acquire information, but also the ecological conditions that favor the opportunities to do so, affects social learning in migrant orangutan males. Thus migrant males are likely balancing a trade-off between socially learning new information from local orangutans, while simultaneously avoiding increased levels of competition with non-related conspecifics in a challenging phase of their lives.^89^ These results complement other studies that found contrasting evidence for effects of ecological conditions on rates of innovations.^22^^,^^23^ In combination, these findings strongly suggest that both ecological “necessity” and “opportunity” can modulate individuals’ social and individual learning. As such, these studies underline the importance of the effects of ecological conditions on cultural transmission. We propose to expand the existing framework on how ecology, specifically food availability, affects social learning and knowledge transmission between adult, non-related individuals, by also incorporating the effects of ecological “necessity” and “opportunity” on the propensities for social learning, rather than solely focusing on actual individual learning occurrences.^31^^,^^70^^,^^71^

The differences in peering rates between the two populations persisted after controlling for differences in food availability. Similar differences in propensities for observational social learning have been found between immatures from the same study populations: immature orangutans in Suaq peer significantly more than their counterparts in Tuanan,^90^ even after controlling for the known differences in association time between the species.^68^^,^^83^^,^^86^^,^^88^ These differences in social learning opportunities and propensities could be the result of either developmental effects, i.e., a consequence of growing up under different ecological conditions (see in the following), or of genetic differences between the species that have split approximately 674 kya ago,^91^ or a combination of both.^92^

Higher rates of individual learning, as evident in more frequent exploratory behavior, have previously been documented in the more sociable population at Suaq compared to the less sociable population at Tuanan.^90^ As a likely result of the higher individual and social learning performances in Suaq, individuals show larger sets of learned skills and overall more learning-intense diets (i.e., a diet that requires more intense pre-ingestive processing), compared to Tuanan.^90^ The results of these studies in combination with the results of the current study are in line with the developmental version of the cultural intelligence hypothesis. The cultural intelligence hypothesis states that growing up with increased opportunities for social learning will first lead to higher *individual* learning abilities, and second to higher *social* learning abilities, through the larger sets of affordances that individuals can draw from when being confronted with a novel problem.^93^ Indeed, in the same populations as studied here, immature orangutans' exploration rates (with exploration being a measure of individual learning ability) are correlated with past experienced sociability during the first years of their lives but were not affected by prevailing food availability.^94^ When housed under comparable conditions in zoos, where food availability is high and stable, Sumatran orangutans outperform their Bornean counterparts in problem-solving tasks.^92^ These differences in cognitive performance despite similar developmental and immediate conditions suggest that the differences in individual and social learning abilities between Sumatran and Bornean orangutans are at least to some degree genetically fixed (evolutionary version of the cultural intelligence hypothesis).

Our finding of frequent opportunities and high propensities for social learning in migrants may affect their cognitive and social learning abilities. Applying the predictions of the developmental effects of social learning opportunities on learning abilities to migrants, who experience frequent and diverse social learning opportunities when, e.g., traveling through unfamiliar habitats, one would predict that migrating individuals may show advanced learning capacities, in terms of social and individual learning abilities. In fact, when human brains are repeatedly exposed to new environments, lasting physical changes like an increase in hippocampal gray matter density can be measured. This is predicted to stand in connection to spatial cognition,^95^ which is significantly increased in individuals that have higher demand for using the brain repeatedly in navigation contexts. Additionally, psychological changes can occur, like an increase in overall social tolerance and empathy, through witnessing other cultures and lifestyles when traveling.^96^^,^^97^ In humans this can lead to knowledge spillovers driven by migrants in multicultural groups, which can increase productivity and innovativeness and eventually stimulate long-lasting cultural diversification.^45^^,^^98^^,^^99^

Discovering effects of prevailing food availability on social tolerance and social learning in the least sociable great ape species that is most distantly related to humans points toward a deep evolutionary origin of ecological effects on social learning and learning propensities in the hominid lineage^100^ and their potential presence in other lineages. All great apes, but also early hominins, such as *Australopithecus africanus* and *Paranthropus robustus*,^101^^,^^102^ show varying forms of sex-biased dispersal^48^^,^^86^ and thus show patterns of migration during the lives of the individuals of the dispersing sex. We thus would expect evolutionary continuity in the ability of migrating individuals to use social learning from resident individuals to adapt to new habitats, when environmental conditions are favorable for mutual knowledge exchange.

Based on the results of our study, we hypothesize that possessing two specific traits provided a survival advantage to migrating individuals in our hominid lineage. First one is the ability to decide whether or not and how close to associate with an unfamiliar conspecific under the prevailing ecological conditions, given that associations bear potential risks and that these risks increase with increasing physical proximity.^36^^,^^40^^,^^41^ Favorable ecological conditions would allow then for close associations and subsequent high-fidelity social learning which could lead to valuable knowledge exchange.^45^^,^^46^^,^^99^ However, during times of scarcity, it may pay off more to avoid competition and disease transmission by increasing physical distance.^44^ Secondly, natural selection likely favored individuals with an increased social learning propensity, which likely improved their overall learning performance which then again allowed for more complex innovations to emerge, such as material technologies during hominin evolution.^10^^,^^103^ Thus, resource availability likely affects the developmental and genetic foundations underlying the propensity to attend to ecological information accessible through local residents, which over time played out to be a pivotal role in the history of human migration and the emergence of our unmatched culture.^10^^,^^12^^,^^104^^,^^105^

### Limitations of the study and future work

Although the difference in migrant peering rates between the two studied populations persisted after controlling for differences in prevailing food availability, with this study, we are unable to differentiate between the developmental and genetic effects on the migrant orangutans’ social learning propensities. This will remain challenging because, e.g., relocation and cross-fostering experiments that would allow to disentangle innate from learned behaviors during ontogeny, are not feasible in great apes like orangutans, for several reasons, including ethical and conservation concerns.

Further, we did not explore the potential of alternative functions of peering behavior, e.g., that peering might be used as a social tool for migrant males, to get access to local females. In a recent study,^106^ it was reported that dispersing male vervet monkeys (*Chlorocebus pygerythrus*) are capable to adjust their levels of sociality to the sociality of the group they migrate to, underlining the importance of social integration after dispersal. Orangutans females are thought to have a certain degree of leverage over males.^107^ Therefore, peering behavior, which also resembles infantile behavior since it is most commonly observed in immatures,^31^ might be one way for males to advertise non-threatening behavior and thus be allowed close by. Whereas this and other potential functions do not negate the possibility of knowledge transfer and thus learning function of peering, it may explain remaining variation in our dataset (see for potential social functions of peering^108^). Indeed, for all analyses shown in the paper, the confidence intervals are larger for the Suaq than the Tuanan population, which means that our model explains less of the variation in the dependent variable (peering probability) in Suaq than in Tuanan. This might correspond to the higher levels of sociability in the Suaq population (including larger and more frequent associations) which may mean that other factors that we did not include in our model (such as social factors) affect peering probabilities in the Suaq population. Especially social factors may have a more pronounced effect on peering at Suaq, the more sociable population.

Another limitation of our study is that at both populations the geographic origins of the migrant males are unknown, and thus it is not known if, and to what extent, their natal areas' habitat differs from the habitats in Suaq or Tuanan. Genetic studies suggest long-distance dispersal,^85^ which makes it likely that the natal habitats differ from the host habitats. However, we cannot assess how much an individual male must learn upon arrival. Generally, Suaq is surrounded by different habitat types such as dryland hill forests, and further away higher-altitude forests, whereas Tuanan is surrounded by similar swamp forests and has no direct connection to different habitat types. Thus, the chances that a migrant male grew up in a very different habitat and therefore is highly unfamiliar with the locally available food items is likely higher for Suaq, than for Tuanan. This may also contribute to the larger confidence intervals for Suaq in our model. However, when investigating migrant orangutan males’ peering behavior in the feeding context, migrant males from both populations peer significantly more at orangutans feeding on skill-intense food items, than at easy-to-process ones, and peered significantly more at rare food items (rarely consumed in the local orangutans’ diet) than at common ones.^57^ Further, migrant peering rates would significantly decrease with increasing time spent in the area. These findings suggest that overall, in both populations, migrating individuals need to learn about locally available foods after dispersal.

Additionally, in our study, we used food availability to assess the effects of ecology on social learning. However, many other ecological factors likely affect the behavior of individuals, such as the nutritional composition and monopolization potential of available food, rain, topography, or temperature, which were not targeted by this study.

Finally, the details of how migrant individuals make actual use of the knowledge, which they witnessed through peering behavior, remain to be investigated. Follow-up studies could tackle this question by assessing the development of the migrant males’ individual behavioral repertoires, and the inclusion of new, learned behaviors with increasing time they spent in association with role models, and the overall time spent in the local area.

## STAR★Methods

### Key resources table

**Table:** 

REAGENT or RESOURCE	SOURCE	IDENTIFIER
**Deposited data**		

Repository data https://data.mendeley.com/datasets/fkd6c452w4/1	This paper	Mörchen, Julia (2023), “Data_Mörchen et al. 2023”, Mendeley Data, V1, doi: https://doi.org/10.17632/fkd6c452w4.1

**Experimental models: Organisms/strains**		

*Pongo abelii*	Wild Sumatran orangutans, Suaq Balimbing research station, 03°02’N; 97°25’E, Gunung Leuser National Park, South Aceh, Sumatra, Indonesia	www.suaq.org
*Pongo pygmaeus wurmbii*	Wild Bornean Orangutans, Tuanan Research Station, 02°15’S; 114°44’E, Mawas Reserve, Central Kalimantan, Indonesia	https://www.aim.uzh.ch/en/research/orangutannetwork/tuananorangutanresearchproject.html

**Software and algorithms**		

R 4.3.0	R Development Core Team, 2023;R Foundation for statistical computing	https://www.r-project.org/

### Resource availability

#### Lead contact

Information and requests for resources should be directed to and will be fulfilled by the lead contact, Julia Mörchen (julia_moerchen@eva.mpg.de).

#### Materials availability

No new materials were generated in this study.

#### Data and code availability

• Data have been deposited at Mendeley and are publicly available as of the date of publication. DOI is listed in the key resources table.
• All original code has been deposited at Mendeley and is publicly available as of the date of publication. DOI is listed in the key resources table.
• Any additional information required to reanalyse the data reported in this paper is available from the lead contact upon request

### Experimental model and subject details

We used data collected on a wild population of Bornean orangutans (*Pongo pygmaeus wurmbii*) from 2010 to 2018 at the Tuanan research station (02°15’S; 114°44’E, Mawas Reserve, Central Kalimantan, Borneo, Indonesia), as well as on a wild population of Sumatran orangutans (*Pongo abelii*) from 2010 to 2020 at the Suaq Balimbing research station (03°02’N; 97°25’E, Gunung Leuser National Park,South Aceh, Sumatra, Indonesia). The focus of this study was on orangutan unflanged males (i.e., males without secondary sexual characteristics^109^), who are migrants due to the male-based natal dispersal of the species (see introduction). We here refer to these males as migrant unflanged males or in short migrant males. In total, we had data on 71 migrant males available. Since the geographic origins of the migrant males are unknown, information on their exact ages are not available. We did not include data on flanged males (i.e., males with secondary sexual characteristics) because of their solitary life-style and low involvement in peering activities.^57^ Associations were defined as when an orangutan spends time with another individual within 0 to 50 meters, and close associations as spending time within distances from 0 to 2 meters. For each male, we calculated a peering rate for each individual he was in association with for each day on which the male was either the focal of a follow, or an association partner of a focal individual. In total, our data contained 1384 daily dyadic associations of 578 different dyads from both sites. Males associated in total with 33 adult females, 55 immatures and 52 unflanged males.

#### Institutional permission information for higher vertebrate model

This study on wild orangutans was strictly observational and non-invasive, and there was no interaction with our study animals in any way. The research protocols were approved by the Indonesian State Ministry for Research, Technology and Higher Education (RISTEK398/SIP/FRP/E5/Dit.KI/X|/2017) and complied with the legal requirements of Indonesia.

### Method details

#### Data collection

We followed a standardized behavioural observation protocol during nest to nest follows of focal individuals (see protocol here: https://www.ab.mpg.de/571325/standarddatacollectionrules_suaq_detailed_jan204.pdf).We included data from 68 observers who conducted instantaneous scan sampling at two-minute intervals on the focal animal's activity as well as the distances of other individuals present. All observers underwent extensive data collection training upon arrival at the stations and achieved at least an 85% agreement level when compared to experienced observers. We collected all occurrence data on peering behaviour at both sites, from focal orangutans and party members. Peering was defined as “the peerer is directly looking at the action of another individual (peering target), sustained over at least 5 seconds, and at close enough range that enables the peering individual to observe the details of the action (within 2 meters in the feeding- and within 5 meters in the nest-building context). The peering individual faces the peering target and shows signs of following the actions of the peering target by head movements, which indicates attentive interest in the action of the target.”, following,^31^ Table 1, page 89. The males peered at a broad variety of behavioural contexts, but most peering was directed at foraging behaviours.^57^ To account for differences in peering duration, we weighted the peering events according to their duration (see Supplement, Table S3 for details). For each peering event, we recorded the identity of the peerer and the peering target. In 88 daily dyadic associations we had at least one peering event (range: 1 – 21) and in 1296 daily dyadic associations there were no peering events, with a total number of 360 individual peering events recorded (see data overview in Supplement, Table S2 for more details). To investigate the impact of temporal variations in food availability on social learning at both sites, we used the fruit availability index (FAI), which we calculated each month as the percentage of fruiting trees in established phenology plots.^62^^,^^110^ Within these plots, all trees with a diameter of >10 cm at breast height (DBH) were checked by trained observers for the presence of fruits. In Suaq the plots contain around 1000 stems and at Tuanan around 1900 trees^62^ and at both sites’ plots are crossing the study area from South to North and West to East. For Suaq the FAI values ranged from 3.89 (minimum) to 17.40 (maximum), with a mean FAI value of 10.01. For Tuanan the FAI values ranged from 0.27 (minimum) to 10.05 (maximum), with a mean FAI value of 3.76.

### Quantification and statistical analysis

The analysis and graphs were done in R, version 4.3.0.^111^ To test if male peering rates increases with increasing FAI and increased when in close distance to the target (prediction 1), and if males in the more sociable population in Suaq peer more than males in the less sociable population of Tuanan (prediction 2), we used a generalized linear mixed model (GLMM) with Poisson family distribution, as implemented in the glmmTMB package.^112^ We included the males` peering count for each dyad as response variable, FAI, time in close association with the dyad partner, and site as predictors, as well as time in association with the dyad partner as an offset term. We further controlled for the dyad id, year, and the peering targets` age sex class, by including them as random effects. Additionally, based on previous results,^57^ we included the random slopes of the absolute number of months a male has been seen in the area, over the random effects. The FAI was z-standardized across sites. We conducted full-null model comparison using likelihood ratio tests (LRT), using the ‘anova’ function, with the null model containing the random effects and the control variables only.^113^ We assessed the effect of each predictor in the full model using the ‘drop1’ function of VGAM package.^114^ We tested the model for overdispersion^115^ and zero-inflation using the DHARMa package.^116^ We obtained a dispersion parameter of 1.41 and zero-inflation ratio of observed to predicted zeros of 1.15, suggesting no dispersion or zero-inflation issues. We validated model stability using influence diagnostics of the “glmmTMB_stability” function provided by,^115^ see full model output in Supplement, Table S1. We assessed the overall fit of the models by calculating the conditional pseudo delta *R*^2^ using the MuMln package.^117^^,^^118^ We detected no influential cases, and no multicollinearity among fixed effects, using the variance inflation factor function from the car package,^113^ with the minimum vif of 1.36 and a maximum of 3.42. We interpreted significant p-values as p < 0.05. We use “ggpredict” function from the ggeffects packages^119^ for each predictor variable separately, in order to plot the predictions and confidence intervals generated by the model, with all other variables respectively were held constant at their mean.
