# Supplementary material for: Orangutan males make increased use of social learning opportunities, when resource availability is high
Source: iScience. 2024 Jan 18;27(2):108940. doi: 10.1016/j.isci.2024.108940 (PMC10850741; doi:10.1016/j.isci.2024.108940)
Supplement: Document S1. Tables S1–S3 [file mmc1.pdf]

iScience, Volume *27*

Supplemental information

Orangutan males make increased use of social learning opportunities, when resource availability is high

Julia Mörchen, Frances Luhn, Olivia Wassmer, Julia A. Kunz, Lars Kulik, Maria A. van Noordwijk, Puji Rianti, Tri Rahmaeti, Sri Suci Utami Atmoko, Anja Widdig, and Caroline Schuppli

Supplemental information

Supplementary Tables

Table S1. Effects on migrant orangutan males peering rates, related to Figure 1, Figure 2, Figure 3 and to Table 1 and Table 2.

The results of the GLMM with Poisson family distribution, analyzing the effects of site, FAI, and time spent in close distance of 2 m to the peering target, on migrant orangutans` peering behavior. Model estimates, standard errors (SE), confidence intervals (CI), Chi-square (χ^2), min. and max (model stability test) and degrees of freedom (df) are provided, based on N = 1384 daily dyadic observations of migrants associating in distances of 0 to 50 m with peering targets. The conditional pseudo delta R^2 was 0.85. Significant p-values are marked in bold font.

N	Response variable	Factor	Factor type	Estimate	SE	Lower CI	Upper CI	χ^2	*df*	*P*	Min	Max	Disp	Family
1384	Migrant males` peering counts at targets	Intercept	Intercept	-0.83	0.22	-7.33	-6.49	-	-	**< 0.001**	-10.04	-5.71	1.41	Poisson
		**Site (Tuanan)**	**Predictor**	-1.4	0.37	-2.16	-0.78	14.63	1	**< 0.001**	-2.63	1.75		
		**FAI**	**Predictor**	0.37	0.16	0.1	0.62	4.45	1	**0.0181**	0.15	0.6		
		**Time within 2 m**	**Predictor**	0.005	0.002	0.004	0.007	9.08	1	**0.0016**	0.003	1.02		

Table S2. Data overview and sample sizes. Ufm = Unflanged male, related to EXPERIMENTAL MODEL AND SUBJECT DETAILS in the **STAR Methods.**

Description	Suaq N	Tuanan N	Total N
Daily dyadic observations	562	822	1384
Dyad combinations	267	311	578
Daily dyadic observations, including peering events	48	40	88
Daily dyadic observations, with peering events = 0	514	782	1296
Ufm (Individuals)	46	25	71
Ufm (Individuals)with peering event	18	15	33
Ufm, no. Of total peering events	298	62	360
Adult female (Individuals)	16	17	33
Immatures (Individuals)	25	30	55
Observation years	10	8	18
Years of data collection	2010-2020	2010-2018	-

Table S3. Method of weighting peering counts depending on peering duration and available information, related to METHOD DETAILS, Data collection, in the **STAR Methods.**

Unit	Time (min)	Time (sec)	Time (sec)	Count
NA				1
UNK				1
15:56 (e.g.)				1
btw. bouts			<120 sec	1
	< 1 min	Minimum 5-60 sec		1
			60 sec	1
	< 2 min	60-120 sec		1
1 bout	2 min		120 sec	2
		120-240 sec		2
2 bouts	4 min		240 sec	3
		240-360 sec		3
3 bouts	6 min		360 sec	4
		360-480 sec		4
4 bouts	8 min		480 sec	5
		480-600 sec		5
5 bouts	10 min		600 sec	6
		600-720 sec		6
6 bouts	12 min		720 sec	7
		720-840 sec		7
7 bouts	14 min		840 sec	8
